# Wilson’s disease causing acute-onset of optic neuropathy in a child: a case report and literature review

**DOI:** 10.1097/MS9.0000000000001770

**Published:** 2024-01-29

**Authors:** Ammar Albostani, Hamza Warda, Fares Alhashemi, Manar Rajabieh, Mohammed Abdulrazzak, Muhamad Kanjo

**Affiliations:** a Faculty of Medicine, University of Aleppo, Aleppo; b Faculty of Medicine, Al-Baath University, Homs, Syria

**Keywords:** case report, Kayser–Fleischer rings, optic neuropathy, Wilson’s disease

## Abstract

**Introduction and importance:**

Wilson disease (WD) is a rare genetic disorder with a wide range of clinical manifestations, including hepatic, neurologic, and psychiatric symptoms. To date, there have been five cases (including our case) representing optic neuropathy caused by WD.

**Case presentation:**

A 15-year-old female presented to the emergency department with neurological symptoms. The patient exhibited confusion but maintained stable vital signs, and physical examinations were all normal. Abdominal ultrasound and axial brain computed tomography (CT) scan were both normal. The patient’s neurological condition and laboratory test results suggested diagnoses of WD. After 2 days, the patient experienced sudden bilateral blindness. The patient’s condition deteriorated rapidly, and was subsequently referred to the ICU.

**Clinical discussion:**

The incidence of the disease varies by ethnicity, with a higher prevalence in Eastern Asian populations. Diagnosis can be challenging due to the diverse presentation of symptoms, but it is important to consider WD as a differential diagnosis in patients presenting with acute hepatitis and/or neurologic abnormalities.

**Conclusion:**

Healthcare professionals should be educated about the diverse clinical manifestations of WD to help in early recognition and diagnosis of the disease.

## Background

Highlights

- This case report presents a rare occurrence of acute bilateral blindness in a young female patient diagnosed with Wilson’s disease.
- Ophthalmic examination revealed normal reactive pupillary, and dilated pupils, with no optic disc oedema characteristics, and Kayser–Fleischer rings have not been noticed.
- Until this moment, there have been five cases (including our case) representing optic neuropathy caused by Wilson disease.

Wilson’s disease (WD), also known as hepatolenticular degeneration (HLD), is an autosomal recessive condition caused by mutations in the ATP7B gene on chromosome 13, which encodes ATP7B protein. This protein is responsible for copper-transporting from inside body cells to the secretory pathway for excretion or incorporation into ceruloplasmin^1,2^.

Recent studies on Eastern Asian populations have shown a higher prevalence of WD than previously thought^3^. The disease typically manifests in children and adolescents, with onset occurring between ages 5 and 35 years^4^. Initially, patients remain asymptomatic but gradually accumulate copper, leading to subclinical liver disease.

Symptoms of WD are categorized into three main groups: hepatic, neurological, and psychiatric. WD primarily affects the nervous system and psychiatric function, with neurological symptoms arising from basal ganglia dysfunction and mental health disturbances. While Kayser–Fleischer (KF) rings in the eyes are uncommon, optic neuropathy resulting in reduced vision has been reported. Treatment options include copper chelators and zinc salt^5,6^.

This case report presents a rare occurrence of acute bilateral blindness in a young female patient diagnosed with WD, providing valuable insights into the neurological development and highlighting the diagnostic challenges associated with such cases.

## Case presentation

A 15-year-old female presented to the emergency department with symptoms of unstable mental state, disorientation, confusion, colicky abdominal pain, and constipation. She had seen a village doctor who attributed her symptoms to viral hepatitis A, and prescribed painkillers. However, her condition did not improve. Her parents reported a family history of spontaneously resolved jaundice with her two younger siblings, and denied any illicit drug use, smoking, alcohol consumption, or significant family history of diseases; furthermore, the patient had no history of vision impairments.

**Figure 1:**
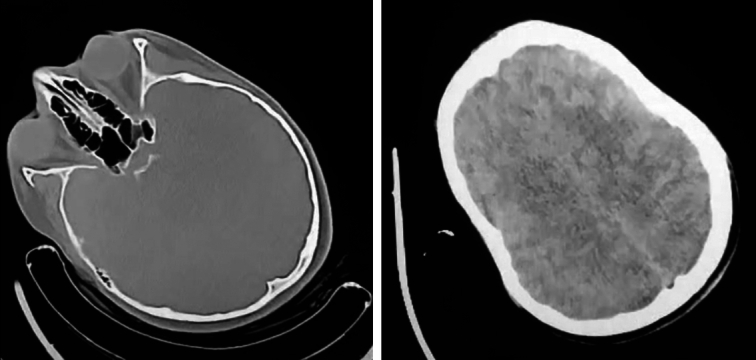
Patient’s brain computed tomography scan revealing normal tissues with no radiological abnormal findings.

Upon admission, the patient exhibited confusion but maintained stable vital signs, including a body temperature of 36.5°C, heart rate of 80 beats per min, respiratory rate of 15 breaths per min, and blood pressure of 135/80 mmHg. Physical examinations of the heart and lungs revealed no abnormalities, her abdomen was normal without hepatomegaly or splenomegaly. Laboratory tests showed elevated urinary copper and decreased serum ceruloplasmin levels, along with abnormally elevated liver enzyme levels (Table 1). Abdominal ultrasound and axial brain computed tomography (CT) scan were both normal (Fig. 1). Due to the patient’s intolerance, a liver biopsy could not be performed. The patient’s neurological condition and laboratory test results suggested diagnoses of WD.

**Table 1:** Patient’s laboratory tests revealing the typical Wilson’s disease findings

Test	Results	Ref. range
Ceruloplasmin	12.6 mg/dl	22–61 mg/dl
Urinary copper (24 h)	127.8 µg/24 h	Up to 60 µg/24 h
Urine volume	1700 ml/24 h	1000–2000 ml/24 h
Total protein	5.5 g/dl	6.0–8.0 g/dl
Total bilirubin	31.5 mg/dl	0.2–1 mg/dl
SGPT (ALT)	278 U/l	Up to 41 U/l
SGOT (AST)	204 U/l	Up to 40 U/l
Alkaline phosphatase ALP	232 U/l	Up to 105 U/l
Anti-HAV (IgM)	9.14 Ru/ml	Up to 1.1 Ru/ml
INR	4.2	0.8–1.1
Prothrombin	22.7 se	11–13.5 s
ANA	0.37	Negative Up to 0.8
Coombs direct	Negative	Negative
Coombs indirect	Negative	Negative

ALP: Alkaline Phosphatase; ALT: Alanine Aminotransferase; AST: Aspartate Aminotransferase; SGPT: Serum Glutamic Pyruvic Transferase; SGOT: Serum Glutamic-Oxaloacetic Transaminase;ANA: Anti-Nuclear Antibodies; INR: International normalized ratio

Less than 2 days after admission, the patient experienced sudden bilateral blindness. Ophthalmic examination revealed normal reactive pupillary, and dilated pupils, with no optic disc oedema characteristics. In addition, KF rings have not been noticed. Due to associated neurological risks, we decided not to administer Penicillamine to the patient. Unfortunately, the patient’s condition deteriorated rapidly, and was subsequently referred to the ICU, the patient passed away on the next day due to acute liver failure.

## Discussion

The first paper that described such case was written by Samuel Alexander Kinnier–Wilson in 1911^7^. Recent mass screening studies suggest that the approximate worldwide incidence rate of WD is 1/30 000–1/100 000, and the disease-causing gene carriers are accounting for 1/90 of population^4^. For ethnic reasons, the incidence rate of WD increases to 1/1500–1/3000 in Eastern Asian people, and in the United Kingdom where the estimated prevalence of WD is 1/7000^8^. Thus, there have been five cases (including our case) representing optic neuropathy caused by WD^1,3,6,9^. (Table 2). The five reported cases included three males and two females, with ages ranging from 14 to 46 years. It is noteworthy that in medical literature the origin of the five case reports presenting WD with optic neuropathy are all Asian.

**Table 2:** Summary of the reported cases of Wilson’s disease presenting with optic neuropathy (Updating for a similar table existed in Yang *et al*.(1) case report)

Study	Age (year)	Sex	Population	Binocular monocular	Onset form	Neurological manifestations	Hepatic manifestations	Geniting testing	Treatment	Outcome
Our Study	15	Female	Caucasian	Binocular	Hepatic	Yes	Acute Hepatitis	Not Tested	No Treatment Induced	Complete blindness and death
Yang *et al.* ^3^	22	Male	Chinese	Binocular	Neurologic	Yes	Early-Stage Cirrhosis	C.3089G>A, P.G1030D; C.2924C>A, P.S975Y	Penicillamine, Zinc, Dmps	Incomplete recovery
Chou *et al.* ^6^	20	Female	Caucasian	Monocular	Hepatic	No	Acute Liver Failure	Not Tested	Liver Transplant	Incomplete recovery
Rukunuzzaman *et al.* ^9^	14	Male	Bengalese	Monocular	Neurologic	Yes	End Stage Liver Disease	Not Tested	Durg Treatment	Complete recovery
Gow *et al.* ^1^	46	Male	Caucasian	Binocular	Ophthalmology	No	Advanced Cirrhosis	Not Tested	Penicillamine	Legal blindness without recovery

Clinical manifestations may occur as early as 8 months in children or as old as 70 years in adults, and for uncertain reasons. Two-thirds of WD patients who experience the onset of their symptoms after the age of 40 initially present with neurological symptoms. On the other hand, the majority of children with WD typically exhibit hepatic symptoms^2,4,8^.

Due to the variety of clinical characteristics, the diagnosis of WD should not be ignored as a differential diagnosis in young patients presenting with acute hepatitis and/or rapid onset of jaundice and haemolytic anaemia^2^. Clinically, liver disease in WD may occur up to 10 years before the onset of neurologic manifestations. Additionally, the majority of WD patients with neurologic symptoms present with liver disease^2^. Therefore, the diagnosis of WD should be considered when a patient presents with hepatic symptoms accompanied by neurologic abnormalities^1^. In our case, we encountered biochemical liver abnormalities in the context of acute hepatitis, in addition to a normal abdominal ultrasound (US) examination, with the absence of hepatomegaly or splenomegaly, decreasing the possibility of a cirrhosis.

The majority of WD patients presenting with neurologic features will have one or more of these manifestations: dysarthria as the most common symptom (57.6%), dystonia (42.4%), unsteady gait (37.8%), irregular, jerky, and dystonic tremors (36.2%), parkinsonism (17.3%), athetosis (15.3%), seizures (4.7%), and ataxia in seldom cases^8,10^. According to EASL Clinical Practice Guideline in WD^2^, life expectancy is higher in patients who present primarily with neurologic symptoms rather than hepatic ones. However, neurologic manifestations are generally considered irreversible. Psychiatric symptoms usually include changes in personality, abnormal behaviour, depression, anxiety, and irritability^8,10^.

In our case, the patient represented with a combination of neuropsychiatric symptoms, including choreic movements, personality changes and unstable mental state, disorientation and confusion. However, only two of the four previous cases (Yang *et al*.^3^ and Rukunuzzaman *et al*.^9^), had neurological symptoms besides optic neuropathy. Hepatic manifestations differ in the five cases, advanced liver diseases have been confirmed in (Gow *et al*.^1^ and Rukunuzzaman *et al*.^9^) patients, while acute liver failure and early-stage cirrhosis in the rest (Chou *et al*.^6^ and Yang *et al*.^3^) was reported.

Acute-onset visual impairments are believed to take place when severe hepatic and/or neuropsychiatric symptoms are not treated^11^. Nevertheless, associated acute bilateral optic neuropathies with WD are rarely reported in the literature^1^. KF rings are considered the hallmark ophthalmological sign in neurologic WD, but are not specific to the diagnosis due to their presentence in some other obstructive liver diseases such as primary biliary cirrhosis^6,8,10^. KF rings are formed by the deposition of copper in Descemet’s membrane in peripheral cornea, and are noticed in 97.6% of neurologic WD cases^11^. Chevalier *et al.* ^5^ stated that KF rings may be helpful for the follow-up assessment since cornea’s density of copper deposit may indicate the severity of the disease.

There is no definitive diagnostic examination, laboratory test, or clinical symptom that can confirm the presence of WD due to variable results and manifestations. However, hepatic copper quantity may be the most accurate detective exam for WD diagnosis^1,11,12^. This diagnostic dilemma may delay the identification of WD which can cause significant problems^10^. Family history of jaundice and/or neuropsychiatric symptoms may be helpful in considering WD in such patients^8^. In clinical practice, KF rings existence and the decreased level of serum ceruloplasmin (CP) to less than 10 mg/dl provide an important clue to WD diagnosis, but as mentioned before, these manifestations are not reliable to establish the diagnosis as, for instance, CP levels can be low in cases of hepatic insufficiency, coeliac disease, and familial aceruloplasminemia^2,8^. Our patient exhibited jaundice colour, decreased CP levels, increased urinary copper, and neuro-psychological symptoms. We decided to diagnose our patient with WD upon these manifestations: however, the deterioration was exceedingly rapid and the patient was irresponsive due to her neuropsychiatric symptoms that we, unfortunately, could neither carry on a liver biopsy nor MRI.

Delayed treatment of WD can cause serious disabilities within few months, and may even lead to rapid deterioration and death. Thus, survival rate and prognosis depend on the early diagnosis and management with the adherence to drug treatment^2,4^. Once WD diagnosis is made, continuous drug therapy throughout lifetime is necessary since low copper diet fails to control copper accumulation.

The mechanism of optic neuropathy in WD is still not well-defined, therefore the association between them has not been well established^1^. Several drugs are available for managing WD, such as D-penicillamine, trientine, zinc, tetra thiomolybdate, and dimercaprol^2^. As it has been reported, 10–50% of patients treated with D-penicillamine during the initial phase of treatment suffered from worsening in neurological symptoms^2^. Treatment strategies for this rare occurrence may involve a combination of chelation therapy and corticosteroids. Chelation therapy is aimed at reducing the copper overload in the body, while corticosteroids are used to reduce inflammation and swelling in the eyes. In some cases, immunosuppressive agents may also be used to control the autoimmune response responsible for the vision loss. It is important to note that the effectiveness of these treatments may vary depending on the severity and duration of the vision loss. Therefore, close monitoring and timely intervention are crucial for optimal outcomes. However, in our case and due to severe neurological manifestations, we decided not to prescribe D-penicillamine treatment to the patient. Therefore, it is improbable that our patient developed vision impairment by drugs of treatment as seen in other cases with WD. However, we acknowledge that treatment strategies may differ in rare and difficult situations, and further research is needed to determine the optimal approach in such cases^6^.

In addition to the unusual presentation of optic neuropathy in our case, KF rings were absent in multiple fundus examinations. In previous studies, KF rings were believed to exist in almost all WD patients with neurological manifestations by approximate rate of 97.6%^11,12^. However, a later study aimed to investigate this phenomenon and ultimately concluded that a significant number of neurological WD patients did not exhibit KF rings, despite these rings being considered a pivotal diagnostic indicator for neurological WD^12^.

Our case report lacks some diagnostic documentations (e.g. liver biopsy and MRI) which is referred to our patient’s health took a sudden downturn, characterized by a swift deterioration of liver function and mental state. We initiated further investigations to thoroughly understand and evaluate the patient’s situation and started crafting a management plan along with the appropriate treatment. But sadly, the patient passed away during this process.

In conclusion, the incidence of the disease varies by ethnicity, with a higher prevalence in Eastern Asian populations. Diagnosis can be challenging due to the diverse presentation of symptoms, but it is important to consider WD as a differential diagnosis in patients presenting with acute hepatitis and/or neurologic abnormalities. Optic neuropathy is a rare but serious complication of WD, and its association with acute liver failure and early-stage cirrhosis should not be overlooked. Ophthalmological signs such as KF rings can aid in the diagnosis and follow-up assessment of neurologic WD. Overall, this case highlights the importance of recognizing the diverse clinical manifestations of WD and the need for early intervention to prevent irreversible neurological damage.

## Conclusions

Healthcare professionals should be educated about the diverse clinical manifestations of WD, including hepatic, neurologic, and psychiatric symptoms. This will help in early recognition and diagnosis of the disease, leading to timely intervention and prevention of irreversible neurological damage. Moreover, public health initiatives aimed at increasing awareness about WD in high-risk communities, and its clinical manifestations can help in early detection and management of the condition, ultimately reducing the burden of the disease on affected individuals and healthcare systems.

## Ethical approval

Not applicable.

## Informed consent

Written informed consent was obtained from the patient for reporting this case and its associated images. The consent is available for review on request.

## Sources of funding

Not applicable.

## Author contribution

All authors fulfil the authorship criteria because of their substantial contributions to the conception, design, analysis, and interpretation of the data. A.A. administrated the project, analyzed the data and drafted the manuscript. H.W. participated in data acquisition and participated in the manuscript drafting. F.A. and M.R. participated in the manuscript drafting and find resources. M.A. reviewed the study and wrote the final version. M.K. conceived the study, and participated in its design. All authors read and approved the final manuscript.

## Conflicts of interest disclosure

The authors declare that they have no competing interests.

## Research registration unique identifying number (UIN)

Our research study does not involve human subjects.

## Guarantor

Mohammed Abdulrazzak.

## Data availability statement

Not applicable.

## Provenance and peer review

Not commissioned, externally peer-reviewed.

## References

[1] Gow PJ Peacock SE Chapman RW. Wilson’s disease presenting with rapidly progressive visual loss: Another neurologic manifestation of Wilson’s disease? J Gastroenterol Hepatol 2001;16:699–701. 11422628 10.1046/j.1440-1746.2001.02381.x

[2] Ferenci P Czlonkowska A Stremmel W. EASL Clinical Practice Guidelines: Wilson’s disease. J Hepatol 2012;56:671–685. 22340672 10.1016/j.jhep.2011.11.007

[3] Yang S Kuang S Xiao Y. Bilateral optic neuropathy as the prominent manifestation of Wilson’s disease. J Clin Neurol (Korea) 2022;18:492–494. 10.3988/jcn.2022.18.4.492 PMC9262453 35796278

[4] Song W Xin L Wang J. A grading method for Kayser Fleischer ring images based on ResNet. Heliyon 2023;9:e16149. 37234668 10.1016/j.heliyon.2023.e16149 PMC10205591

[5] Chevalier K Mauget-Faÿsse M Vasseur V. Eye involvement in Wilson’s disease: a review of the literature. J Clin Med 2022;11:2528. 35566651 10.3390/jcm11092528 PMC9102176

[6] Chou LT Horkey D Slabaugh M. Acute-onset optic neuropathy in wilson’s disease. Case Rep Ophthalmol 2018;9:520–525. 30687074 10.1159/000495744 PMC6341316

[7] Kinnier Wilson SA Wilson SAK Wilson SAK. Progressive lenticular degeneration: a familial nervous disease associated with cirrhosis of the liver together with an experimental research into the anatomy and physiology of the lenticular nucleus. Published online 1911. Accessed October 15, 2023. https://era.ed.ac.uk/handle/1842/34489

[8] Bandmann O Weiss KH Kaler SG. Wilson’s disease and other neurological copper disorders. Lancet Neurol 2015;14:103–113. 25496901 10.1016/S1474-4422(14)70190-5 PMC4336199

[9] Rukunuzzaman M Karim MB Rahman MM. Wilson’s disease in children with blindness: an atypical presentation-Mymensingh Med J 2013:22;176–9. 23416828

[10] Lorincz MT. Neurologic Wilson’s disease. Ann NY Acad Sci. 10.1111/j.1749-6632.2009.05109.x 20146697

[11] Zheng ZW Xu MH Sun CB. Acute-onset visual impairment in Wilson’s Disease: a case report and literature review. Front Neurol 2022;13. 10.3389/fneur.2022.911882 PMC9237335 35775054

[12] Youn J Kim JS Kim HT. Characteristics of neurological Wilson’s disease without Kayser-Fleischer ring. J Neurol Sci 2012;323:183–186. 23043908 10.1016/j.jns.2012.09.013

